# Biomechanical Analysis of the Breaststroke Kick in Young Swimmers Using Wearable Inertial Sensors: An Exploratory Pilot Study

**DOI:** 10.3390/s26051691

**Published:** 2026-03-07

**Authors:** Denisa-Iulia Brus, Răzvan Sandu Enoiu, Dorin-Ioan Cătană

**Affiliations:** 1 Department of Motor Performance, Transilvania University of Brașov, 500036 Brașov, Romania; razvan.enoiu@unitbv.ro; 2 Department of Materials Engineering and Welding, Transilvania University of Brasov, 500036 Brașov, Romania; catana.dorin@unitbv.ro

**Keywords:** wearable inertial sensors, swimming biomechanics, breaststroke kick, human motion analysis, sensor-based training, sports performance

## Abstract

Breaststroke performance is highly dependent on lower-limb biomechanics and the coordination of movement during the kick cycle. Recent advances in wearable inertial sensor technology enable objective analysis of human motion in real training environments. This study presents an exploratory pilot investigation aimed at evaluating the feasibility of using wearable inertial sensors for biomechanical analysis of the breaststroke kick in young swimmers. Five male children (aged 8–10 years) with basic breaststroke proficiency participated in a single-group pre–post exploratory study conducted over a three-month period. Lower-limb motion was monitored using wearable inertial measurement units attached bilaterally to the shanks and feet, allowing real-time kinematic feedback and data recording during training sessions. The intervention consisted of five structured training sessions integrating drill-based breaststroke kick exercises with sensor-assisted feedback. Outcome measures included time-based swimming performance tests (40 m breaststroke kick with kickboard and 40 m breaststroke without kickboard) and qualitative biomechanical evaluations of the passive and active phases of the breaststroke kick. Additionally, selected IMU-derived kinematic variables (peak ankle dorsiflexion and external foot rotation angles) were analyzed to provide quantitative biomechanical insight. Following the intervention, improvements were observed across all outcome measures, including reduced swimming times and increased technique scores assigned by two independent evaluators. These findings support the feasibility of integrating wearable IMUs for technique monitoring and simple kinematic quantification of breaststroke kick mechanics in young swimmers; larger controlled studies are required to assess efficacy.

## 1. Introduction

Performance in competitive swimming is strongly influenced by the efficiency of movement execution and the swimmer’s ability to generate propulsion while minimizing hydrodynamic resistance. Among the four competitive strokes, breaststroke is considered one of the most technically complex, due to the high coordination demands between upper and lower limbs and the dominant contribution of the lower-limb kick to propulsion [1,2]. Small variations in foot orientation, ankle dorsiflexion, and timing between recovery and propulsion phases can lead to substantial differences in swimming efficiency and race performance [3].

Traditionally, swimming technique analysis has relied on video-based motion capture systems, including above-water and underwater cameras [4,5]. While such methods provide valuable qualitative and quantitative information, they present several limitations, particularly in routine training environments. Video-based systems often require complex setups, specialized facilities, and time-consuming post-processing, which restrict their frequent use during regular training sessions [6]. Moreover, occlusion, refraction effects in water, and limited capture volumes can further reduce measurement accuracy, especially for distal segments such as the feet and ankles [7].

In recent years, wearable inertial measurement units (IMUs) have emerged as a promising alternative for human motion analysis in sports [8,9]. IMUs offer several advantages over optical systems, including portability, ease of use, and the ability to collect data in real training and competition environments without constraining the athlete’s movement [10,11]. In swimming, inertial sensors have been increasingly explored for stroke detection, lap counting, velocity estimation, and technique analysis across different strokes [12,13,14].

Recent literature has strengthened the evidence base for using wearable sensing in swimming, especially for training environments where laboratory-grade optical systems are difficult to deploy. A recent systematic review focusing specifically on real-time feedback wearables in swimming concluded that most validated solutions can provide sufficiently accurate kinematic information for training monitoring, although accuracy and reliability remain highly dependent on sensor placement, algorithms, and the measured variable [15]. Real-time biomechanical feedback systems are increasingly emphasized as an enabling technology for skill acquisition, because they close the loop between sensing, processing, and immediate feedback delivery during the execution of the movement [16,17]. In swimming, this capability is particularly relevant for young athletes, where technique consolidation is a key training priority and timely feedback may accelerate error correction.

In parallel, multiple recent studies have demonstrated that IMU-based approaches can quantify performance- and technique-related metrics under ecologically valid pool conditions. For example, phase-based performance evaluation using a single IMU has been proposed and validated across the main swimming techniques, enabling the extraction of kinematic features associated with push-off, glide, stroke preparation, and free-swimming phases [18]. Validation work has also expanded to additional swimmer populations and contexts, supporting the use of single-IMU solutions for lap time and stroke mechanics estimation when compared against video references. Importantly for the present study, recent breaststroke-specific work has highlighted both the potential and current limitations of inertial sensing for breaststroke velocity characterization, suggesting that IMUs can capture informative patterns but may not be interchangeable with other gold-standard devices depending on the target metric [19]. Collectively, these recent findings justify continued development and feasibility testing of IMU-assisted training interventions aimed at technique monitoring and correction in breaststroke—particularly in young swimmers, where objective feedback may complement traditional coaching cues [20].

Despite growing interest, the application of wearable inertial sensors in breaststroke-specific lower-limb analysis remains relatively limited compared to other swimming strokes. The aquatic environment poses unique challenges for sensor-based measurements, including water resistance, sensor fixation stability, and signal interpretation during cyclic underwater movements [21]. Furthermore, many existing studies focus on adult or elite swimmers, while fewer investigations address young swimmers at early stages of technical development, where motor learning and technique consolidation are particularly critical [22].

Another limitation of current approaches is the predominant focus on post-session analysis, with limited use of real-time feedback during training. Motor learning research suggests that immediate, task-relevant feedback can enhance skill acquisition, particularly in young athletes [23]. Integrating wearable sensor technology with real-time visualization tools may therefore provide additional benefits by supporting technique awareness and facilitating corrective interventions during training sessions.

In this context, sensor-assisted training interventions represent a promising direction for swimming performance development. By combining structured drill-based training with wearable inertial sensors capable of real-time feedback and data recording, it may be possible to accelerate technique refinement while maintaining ecological validity in the training environment.

Therefore, the purpose of this study was to conduct an exploratory pilot investigation examining the effects of a sensor-based training intervention on breaststroke kick performance in young swimmers. Specifically, the study aimed to assess changes in (i) time-based swimming performance and (ii) qualitative execution of the passive and active phases of the breaststroke kick following a short-term training program supported by wearable inertial sensor monitoring.

## 2. Materials and Methods

This section describes the study design, participant characteristics, sensor instrumentation, experimental protocol, performance assessments, and statistical analysis procedures used in this exploratory pilot investigation. All methodological aspects were defined to ensure consistency between baseline and post-intervention measurements and to support the feasibility evaluation of the proposed sensor-assisted training approach.

### 2.1. Study Design and Setting

This study was designed as an exploratory pilot investigation to examine changes in breaststroke kick performance following a sensor-based training intervention. Accordingly, the present work should be interpreted as a feasibility and proof-of-concept study. A controlled pre–post experimental design was employed over a three-month period in an indoor swimming pool, under standardized training conditions. All experimental sessions were performed in the same facility to minimize environmental variability, including pool configuration and training context.

All training and testing sessions were conducted at the same time of day (±30 min) to minimize potential circadian influences on performance and neuromuscular function.

To ensure internal consistency, all testing and training sessions were carried out using identical procedures, drill sequences, and performance instructions. Participants were evaluated under comparable conditions at baseline and post-intervention, with particular attention to maintaining consistent start procedures, swimming distances, and rest intervals. The intervention focused specifically on lower-limb breaststroke kick mechanics, integrating a structured drill-based training program with wearable inertial sensor-based motion monitoring and real-time kinematic feedback to support technique correction.

The study employed a single-group pre–post exploratory design without a control group. Therefore, the findings should be interpreted as preliminary feasibility observations rather than evidence of causal effectiveness of the sensor-assisted intervention.

### 2.2. Participants

A total of five male children participated in this exploratory pilot study (mean age: 8.8 ± 0.84 years; range: 8–10 years). All participants were young swimmers with basic breaststroke proficiency, positioned between beginner and intermediate levels of technical development. The sample was intentionally selected to form a relatively homogeneous group in terms of age and swimming background, thereby reducing inter-individual variability in movement execution.

Participants had a mean body height of 133.4 ± 2.70 cm and a mean body mass of 30.6 ± 2.70 kg. All participants had between 2 and 3 years of structured swimming training experience and trained approximately 2–3 sessions per week (total weekly swimming volume approximately 1300–1500 m). All swimmers were classified as beginner-to-intermediate level based on coach assessment and limited competition exposure.

### 2.3. Instrumentation and Sensor Configuration

Lower-limb kinematics were monitored using wearable inertial measurement units (IMUs) (Movella Xsens DOT, Movella Technologies, Enschede, The Netherlands). The Xsens DOT is a micro-electro-mechanical system (MEMS)-based IMU integrating a triaxial accelerometer, gyroscope, and magnetometer. Sensor orientation is estimated using the manufacturer’s proprietary sensor fusion algorithm, as described in the Xsens DOT User Manual [24].

**Figure 1:**
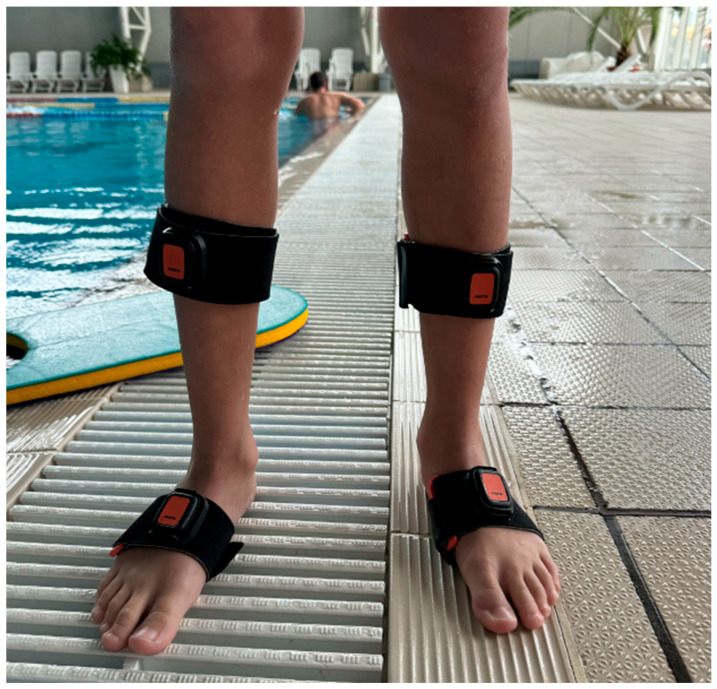
Placement of wearable inertial sensors on the lower limbs. Sensors were attached bilaterally to the shanks and feet to capture segment-level kinematics relevant to breaststroke kick mechanics.

The sensors are IP68-rated and suitable for aquatic environments. Four IMUs were attached to each participant’s lower limbs to capture segment-level motion relevant to breaststroke kick mechanics. Sensors were positioned bilaterally on the shanks and feet, resulting in the following configuration: one sensor on the right shank, one on the left shank, one on the right foot, and one on the left foot (Figure 1).

This placement strategy was selected to capture lower-limb segment orientation and movement patterns associated with ankle dorsiflexion, foot external rotation, and propulsion during the breaststroke kick.

Sensor attachment was performed using secure fixation methods to minimize displacement during swimming activity and repeated push-offs from the pool wall. The same sensor placement protocol was applied consistently across all participants and experimental sessions to ensure comparability between baseline and post-intervention measurements.

The sensor coordinate system follows a right-handed Cartesian reference frame and reports orientation relative to a local East-North-Up (ENU) reference system. Relative joint angles were calculated from the orientation of the foot sensor with respect to the shank sensor.

Ankle dorsiflexion was defined as the relative sagittal-plane rotation between foot and shank segments. External foot rotation was defined as the relative transverse-plane rotation of the foot segment with respect to the shank segment.

Sensors were synchronized prior to each recording session using the Movella multi-sensor root-node configuration to ensure temporal alignment across segments. The sampling frequency was set to 60 Hz.

Two acquisition modes were used:

- real-time streaming via Bluetooth Low Energy 5.0 (BLE) to a tablet device running the KineXYZ application;
- onboard logging in internal memory for offline export (.csv format).

During each training session, motion data were visualized in real time using the KineXYZ application on a tablet positioned poolside within approximately 2–3 m of the swimmer. The platform provides a three-dimensional visualization of lower-limb segments based on orientation data and displays relative joint angles, enabling direct observation of ankle dorsiflexion, foot external rotation, and lower-limb symmetry during drill execution. Although data were streamed in real time, practical feedback delivery occurred primarily immediately after each drill repetition at the pool edge, when the swimmer and coach reviewed the visualization together. Therefore, the system functioned as near-real-time feedback rather than continuous in-water visual guidance. Feedback was reviewed after each drill repetition or short set (typically every 30–60 s), using the 3D avatar and numerical angle readouts (ankle dorsiflexion and foot rotation) displayed by the application.

The same angle outputs were also exported in .csv format for offline extraction of peak values and descriptive analysis.

Because Bluetooth transmission is attenuated underwater, real-time connectivity was most stable when the lower limbs were near the water surface, particularly during the recovery phase of the breaststroke kick. In the event of temporary signal interruption during deeper submersion, onboard logging ensured complete data capture, and data streaming resumed when connectivity was re-established, while onboard logging ensured that complete data were available for offline export. No complete data losses were observed during the analyzed trials.

In addition to real-time visualization, recorded IMU data were exported for quantitative biomechanical analysis. For each testing session (baseline and post-intervention), three consecutive kick cycles performed during the central portion of the 40 m test were analyzed to minimize start and fatigue effects. Peak ankle dorsiflexion and peak external foot rotation values were extracted from the exported orientation data and averaged per participant.

Orientation data were processed using the manufacturer’s embedded sensor fusion algorithm. No additional external filtering was applied.

### 2.4. Sensor Data Acquisition and Processing

The Movella Xsens DOT sensors integrate a triaxial accelerometer, gyroscope, and magnetometer within a MEMS-based architecture. Orientation estimates are computed using the manufacturer’s proprietary sensor fusion algorithm, as described in the Xsens DOT User Manual [24].

Data were recorded at a sampling frequency of 60 Hz. According to manufacturer specifications, the sensors include:

- a triaxial accelerometer (±16 g range);
- a triaxial gyroscope (±2000 °/s range);
- a triaxial magnetometer.

The selected sampling frequency provided adequate temporal resolution for cyclic breaststroke kicking while allowing stable onboard storage and real-time streaming.

Prior to each recording session, sensors were synchronized using the Movella root-node configuration to ensure temporal alignment. Participants adopted a standardized upright anatomical reference posture (standing position, feet parallel, knees extended, arms relaxed) for approximately 5 s to initialize orientation estimation and establish segment alignment relative to the global reference frame.

The default magnetometer-assisted sensor fusion mode was used. Measurements were performed in an indoor swimming pool environment. Although no major ferromagnetic structures were present in the immediate vicinity of the measurement lane, minor magnetic disturbances cannot be fully excluded and are acknowledged as a potential source of measurement error.

Sensors were positioned longitudinally along the shank and dorsum of the foot, aligned visually with the anatomical long axis of each segment. The sensor-fixed coordinate system was therefore assumed to approximate the anatomical segment coordinate frame.

Relative joint angles were computed from the orientation of the foot sensor with respect to the shank sensor. Ankle dorsiflexion was defined as the relative rotation in the sagittal plane, and external foot rotation as the relative transverse-plane rotation between the two segments.

No additional anatomical calibration (e.g., functional calibration trials) was performed, given the exploratory nature of the study and the focus on within-subject pre–post comparison.

Orientation data were processed using the embedded sensor fusion algorithm provided by the manufacturer. No additional external filtering was applied. The use of sensor fusion integrating accelerometer, gyroscope, and magnetometer signals mitigates gyroscopic drift during cyclic aquatic motion.

Because the analyzed variables were peak angles within short kick cycles, long-term drift effects were considered negligible.

The Xsens DOT sensors are IP68-rated and were used without additional waterproof housings. Sensors were secured using elastic fixation bands to prevent displacement during swimming. The system does not involve electrical stimulation or wired connections, and no adverse events occurred during the study.

All procedures were conducted under direct supervision of the coach and investigator to ensure participant safety.

### 2.5. Experimental Protocol and Training Program

The experimental intervention was implemented over a three-month period within the participants’ regular weekly swimming training schedule. The study included five structured sensor-assisted intervention modules that were distributed across this three-month timeframe.

Although five predefined experimental modules were implemented, these sessions were not conducted consecutively over five weeks. Instead, they were spaced periodically across the three-month period to allow consolidation within the normal training process. Between intervention modules, participants continued their standard swimming training program (two to three sessions per week), without additional sensor-based feedback.

The regular weekly training schedule consisted of two to three additional swimming sessions per week (approximately 60 min each), focusing on general stroke technique, aerobic conditioning, and basic skill development. No other technology-assisted interventions were introduced during the study period.

The core of the intervention focused on the correction and consolidation of breaststroke kick technique, with particular emphasis on ankle dorsiflexion, external rotation of the feet, knee separation during the recovery phase, and propulsion efficiency during the active phase of the kick. Training content was progressively structured, beginning with dry-land and poolside drills aimed at developing movement awareness and correct motor patterns, and advancing toward in-water drills performed over increasing distances and under varying breathing conditions.

Early sessions emphasized controlled execution and technical precision, using imitation exercises performed in seated or supine positions and pool-edge drills with partial body support. As the program progressed, participants performed in-water breaststroke kick drills, both with and without a kickboard, integrating head position control and breathing coordination. Constraint-based exercises, such as drills performed with a training stick placed between the legs, were incorporated to promote symmetrical movement and correct limb alignment.

Each session lasted approximately 60 min and included a general warm-up (10–15 min), a targeted breaststroke kick training component (25–30 min), and complementary swimming exercises (15–20 min). The total swimming volume per session ranged between 800 and 1200 m. Rest intervals between individual drill repetitions ranged from 30 to 60 s, while rest intervals between sets ranged from 1 to 2 min. These rest durations were kept constant across sessions and participants. Training intensity was maintained at a low-to-moderate level, emphasizing technical precision rather than maximal effort.

Throughout all training sessions, participants wore the wearable inertial sensors described above. Real-time kinematic feedback provided via the KineXYZ application was used to support technique correction by allowing swimmers and coaches to visually inspect lower-limb movement execution during drills. Sensor-based feedback complemented traditional verbal coaching cues and was used consistently across all sessions.

Training load (i.e., drill duration, distance, and number of repetitions) was standardized across participants and sessions to ensure comparability of exposure to the intervention.

A detailed description of the specific drills performed in each training session is provided in the Supplementary Material.

### 2.6. Performance Tests and Outcome Measures

To evaluate the effects of the sensor-based training intervention, participants completed a battery of four breaststroke-specific performance tests, administered before (baseline) and after (post-intervention) completion of the training program. All tests were conducted under standardized conditions, using identical procedures at both time points.

#### 2.6.1. 40 m Breaststroke Kick with Kickboard

This test assessed lower-limb propulsion efficiency during isolated breaststroke kick execution. Participants started in the water at the pool wall, holding a kickboard with both hands. At an auditory start signal provided by the coach, the participant pushed off from the wall and swam a distance of 40 m using breaststroke kick only, with arms extended on the kickboard.

Each participant performed two trials, and the best time (seconds) was recorded for analysis. Consistent start procedures and rest intervals were maintained across participants and testing sessions.

#### 2.6.2. 40 m Breaststroke (No Kickboard)

This test evaluated overall breaststroke swimming performance without external support. Participants started from the pool wall and swam 40 m breaststroke following the start signal. As with the previous test, two trials were performed, and the fastest time was retained for subsequent analysis.

#### 2.6.3. Passive Phase Technique Evaluation of the Breaststroke Kick

Technical execution during the passive (recovery) phase of the breaststroke kick was evaluated using a criterion-based 10-point ordinal scoring system. Participants performed a 40 m breaststroke kick while holding a kickboard to isolate lower-limb movement.

The following technical aspects were assessed:

- external rotation of the feet
- backward orientation of the soles
- outward rotation of the toes
- ankle dorsiflexion during the recovery phase

Each criterion contributed to a global technique score ranging from 1 to 10, where

- 1–3 indicated incorrect or inconsistent execution of the movement component;
- 4–6 indicated partially correct execution with visible technical deviations;
- 7–8 indicated generally correct execution with minor inconsistencies;
- 9–10 indicated technically correct, stable, and symmetrical execution throughout the kick cycle

Two evaluators (an experienced swimming coach and the investigator) independently rated each participant during baseline and post-intervention testing. Raters were not blinded to the testing time point, which is acknowledged as a potential source of expectancy bias.

For descriptive analysis, scores from both raters were reported separately. Inter-rater reliability was assessed using the intraclass correlation coefficient (ICC), two-way mixed-effects model, and absolute agreement.

Scoring rubric and reliability procedures are detailed in Section 2.6.6 and Supplementary Material S2.

#### 2.6.4. Active Phase Technique Evaluation of the Breaststroke Kick

Technical execution during the active (propulsive) phase of the breaststroke kick was evaluated using the same 10-point scoring procedure described in Section 2.6.3.

The following propulsion-specific aspects were assessed:

- full-foot contact during propulsion
- maintenance of ankle dorsiflexion
- energetic and coordinated movement execution
- quality of the glide phase following propulsion

Scoring was performed independently by the same two evaluators under identical conditions. Inter-rater reliability was quantified using the intraclass correlation coefficient (ICC), two-way mixed-effects model, and absolute agreement.

Scoring rubric and reliability procedures are detailed in Section 2.6.6 and Supplementary Material S2.

#### 2.6.5. Test Standardization

All performance tests were conducted in a 40 m indoor swimming pool under controlled environmental conditions (water temperature: 27–28 °C). Participants used the same lane during baseline and post-intervention testing to avoid external interference. Starts were performed from within the water using a standardized wall push-off without diving. A rest interval of 3 min was provided between repeated trials and 5 min between different test types to minimize fatigue effects.

Time measurements were obtained using manual hand timing with a digital stopwatch (precision 0.01 s), operated by the same experienced coach for both baseline and post-intervention sessions to ensure procedural consistency.

#### 2.6.6. Technique Scoring Procedure and Reliability

Technique quality was assessed using a criterion-based 10-point rubric for the passive (recovery) and active (propulsive) phases of the breaststroke kick. Each phase was evaluated independently by two raters (an experienced swimming coach and the investigator) using the same scoring sheet (Supplementary Material S2). The rubric operationalized the criteria listed in Section 2.6.3 and Section 2.6.4 by defining anchor descriptors for low (1–3), moderate (4–6), good (7–8), and excellent (9–10) execution, based on segment alignment and movement consistency observed during the 40 m kick-with-kickboard trials. Raters scored participants independently; raters were not blinded to the assessment time point (baseline vs. post-intervention), which is acknowledged as a potential source of expectancy bias. Inter-rater reliability was quantified using an intraclass correlation coefficient (ICC; two-way mixed-effects model, absolute agreement). For analysis, rater scores were reported separately and, when a single value was required, the mean of the two ratings was used.

### 2.7. Ethical Considerations

The study was conducted in accordance with the Declaration of Helsinki. According to the national regulations governing non-interventional research in Romania and the institutional policies of Transilvania University of Brașov, formal approval from an Institutional Review Board was not required because the study involved exclusively non-invasive performance monitoring conducted during routine sports training sessions, without medical intervention, biological sampling, or collection of sensitive health data. The procedures implemented did not exceed the normal scope of structured swimming training activities. Written informed consent was obtained from the legal guardians of all participants prior to enrollment.

The study did not involve any experimental manipulation exceeding the normal scope of age-appropriate sports training activities.

### 2.8. Statistical Analysis

For each outcome measure, mean differences between post-intervention and baseline values were calculated to quantify performance changes following the training program. For sensor-derived kinematic variables (peak ankle dorsiflexion and peak external foot rotation angles), mean values were calculated for each participant at baseline and post-intervention. Pre–post differences were reported descriptively as mean ± SD and mean change (Δ). Given the exploratory nature and small sample size, no inferential statistical tests were applied to kinematic variables; instead, emphasis was placed on magnitude and direction of change. Statistical analyses were performed using IBM SPSS Statistics (Version 26.0, IBM Corp., Armonk, NY, USA).

Descriptive statistics are reported as mean ± standard deviation (SD) for baseline and post-intervention values. For each outcome, the paired difference (post–pre) was calculated for each participant, and the mean ± SD of these differences is presented.

Given the exploratory nature of the study and the small sample size (*N* = 5), no formal hypothesis testing was emphasized. Instead, magnitude of change was quantified using paired standardized effect sizes (Hedges g for dependent samples), calculated based on the mean of the paired differences divided by the standard deviation of the differences, with small-sample correction applied.

Individual participant values are provided in the Supplementary Material to enhance transparency.

## 3. Results

This section presents the descriptive results of the time-based performance tests, qualitative technique evaluations, and sensor-derived kinematic variables obtained before and after the intervention. Given the exploratory nature of the study (*N* = 5), results are reported descriptively using mean ± standard deviation (SD) and paired mean differences.

### 3.1. Time-Based Performance Outcomes

#### 3.1.1. 40 m Breaststroke Kick with Kickboard

All participants demonstrated faster completion times following the intervention. Descriptive statistics are presented in Table 1.

Mean completion time decreased by 9.06 ± 1.69 s from baseline to post-intervention. The magnitude of change corresponded to a very large standardized effect size (Hedges g = 4.79). Individual participant values are provided in the Supplementary Material.

#### 3.1.2. 40 m Breaststroke (No Kickboard)

Performance also improved in the full-stroke condition (Table 2).

Mean swimming time decreased by 8.17 ± 1.28 s following the intervention. The standardized magnitude of change was very large (Hedges g = 5.30). Individual values are provided in the Supplementary Material.

### 3.2. Technique Evaluation Outcomes

Inter-rater reliability was good for both qualitative technique outcomes. For the passive phase, ICC(A,1) = 0.774 (ICC(A,2) = 0.873). For the active phase, ICC(A,1) = 0.787 (ICC(A,2) = 0.881).

#### 3.2.1. Passive Phase Technique Scores

Results based on the mean of the two raters are presented in Table 3.

Passive phase scores increased by 2.05 ± 0.68 points, corresponding to a large standardized effect size (Hedges g = 2.72).

#### 3.2.2. Active Phase Technique Scores

Results for the active phase are presented in Table 4.

Active phase scores increased by 1.35 ± 0.74 points, representing a large standardized effect size (Hedges g = 1.54).

### 3.3. Sensor-Derived Kinematic Variables

Peak ankle dorsiflexion and peak external foot rotation angles were extracted from IMU orientation data and averaged across three kick cycles per participant. Results are presented in Table 5.

Peak ankle dorsiflexion increased by 6.2 ± 0.84°, while peak external foot rotation increased by 7.2 ± 0.45°. Both variables demonstrated very large standardized effect sizes.

### 3.4. Summary of Observed Changes

Across all outcome measures, consistent improvements were observed from baseline to post-intervention. These findings indicate substantial changes in performance time, qualitative technique scores, and selected sensor-derived kinematic parameters within this small exploratory cohort. Given the absence of a control group and the limited sample size, these results should be interpreted as descriptive magnitude indicators rather than causal evidence of intervention efficacy.

Standardized effect sizes should be interpreted cautiously. In very small samples (*N* = 5), standardized estimates such as Hedges g are statistically unstable and may overestimate magnitude. Therefore, effect sizes are reported solely as descriptive indicators of change and not as inferential measures of intervention efficacy.

Extremely large values observed for some kinematic variables reflect low within-subject variability rather than large population-level effects.

## 4. Discussion

The present exploratory pilot study aimed to examine the feasibility and potential effects of a sensor-assisted training intervention on breaststroke kick performance in young swimmers. The principal findings indicate consistent improvements across both time-based performance measures and qualitative evaluations of passive and active kick phases following the intervention. These results suggest that integrating wearable inertial sensors with structured technical drills may facilitate measurable improvements in lower-limb coordination and propulsion efficiency in youth swimmers.

### 4.1. Performance Improvements in the Context of Breaststroke Biomechanics

The observed reductions in completion time in both the isolated kick condition and full-stroke breaststroke condition are biomechanically meaningful. Previous biomechanical investigations have emphasized the dominant contribution of lower-limb propulsion in breaststroke, particularly during the propulsive phase characterized by coordinated hip, knee, and ankle extension [3,19,20]. Small improvements in segment alignment and timing can produce disproportionate gains in propulsion efficiency and stroke economy.

The quantitative sensor-derived analysis further supports these findings. Increases in peak ankle dorsiflexion and external foot rotation angles were observed following the intervention, indicating improved segment positioning during both the recovery and propulsive phases of the kick. These kinematic adaptations are consistent with biomechanical principles emphasizing optimal foot orientation and ankle positioning for effective breaststroke propulsion.

In the present study, peak ankle dorsiflexion increased by 6.2°, while external foot rotation increased by 7.2° following the intervention. Although prior biomechanical studies have primarily examined propulsion efficiency and intracycle velocity variations rather than specific ankle orientation magnitudes [3,19], they consistently highlight the importance of appropriate foot alignment during both recovery and propulsion phases. The direction of the kinematic changes observed here is therefore consistent with established mechanical models of breaststroke propulsion, which emphasize sagittal-plane ankle positioning and transverse-plane foot orientation as contributors to effective propulsive surface alignment [3].

Although most IMU-based swimming studies have focused on stroke detection and velocity estimation rather than training interventions, recent work has demonstrated that phase-specific coordination metrics derived from wearable sensors are associated with performance level and technical consistency [16,17,18]. The direction and magnitude of improvements observed in the present study are therefore coherent with current biomechanical understanding of breaststroke propulsion and support the relevance of lower-limb-focused technique refinement.

Importantly, the larger relative improvement observed in the kick-only condition compared with the full-stroke condition is consistent with the hypothesis that targeted lower-limb intervention may be associated with changes in propulsion mechanics, independent of upper-limb contribution. The approximately 9 s improvement observed in the isolated kick condition suggests that modifications in lower-limb mechanics may have contributed meaningfully to performance enhancement. While direct propulsion force was not measured, the previous literature indicates that small improvements in segment coordination and timing can produce measurable gains in stroke efficiency [2,3], which is compatible with the magnitude of change observed in the present cohort.

### 4.2. Wearable Inertial Sensors in Swimming Training Environments

In recent years, wearable inertial measurement units have gained increasing validation for aquatic applications, including stroke phase segmentation, lap detection, and velocity profiling [6,8,10,14]. Systematic reviews have reported satisfactory reliability under ecological pool conditions, while emphasizing the importance of sensor placement and algorithmic processing [6,10].

The present study contributes to this evolving body of literature by applying IMUs not solely as passive monitoring devices, but as active real-time feedback tools integrated directly into the training process. While previous research has validated IMU-derived metrics against video or reference systems, fewer studies have investigated the pedagogical implications of embedding wearable sensing within structured technical interventions. In this respect, the current findings extend the technological application of IMUs from measurement toward technique-oriented training support, particularly in a youth population. To our knowledge, few studies have examined short-term sensor-assisted technique interventions in youth breaststroke swimmers under ecological training conditions.

Furthermore, breaststroke-specific IMU research remains comparatively limited relative to front crawl investigations. Recent breaststroke-focused analyses have demonstrated that inertial sensing can capture meaningful kinematic patterns, although limitations remain in relation to gold-standard velocity systems [14]. In contrast to studies primarily focused on stroke segmentation or velocity estimation [18,19], the present investigation demonstrates that IMU-derived joint orientation metrics are sensitive to short-term pre–post technical modifications. This finding supports the feasibility of extending IMU applications beyond monitoring toward structured technique refinement in youth swimming populations.

### 4.3. Real-Time Feedback and Motor Learning in Young Athletes

A distinctive aspect of the present intervention was the integration of real-time kinematic visualization via the KineXYZ platform. Motor learning theory emphasizes the importance of augmented feedback, particularly during early stages of skill acquisition. Immediate visual feedback can enhance movement awareness, reduce reliance on delayed verbal instruction, and support more rapid error correction. The observed increases in qualitative technique scores (Δ 2.05 points for the passive phase and Δ 1.35 points for the active phase) are compatible with augmented feedback models described in motor learning literature [23], which propose that externally provided visual information enhances error detection and movement recalibration. Although causality cannot be established within the present design, the pattern of improvement aligns with these theoretical frameworks.

In swimming contexts, most wearable-based analyses are performed post-session. By contrast, the real-time visualization employed in the present study enabled a closed-loop feedback system during drill execution. For young swimmers (8–10 years), who are still consolidating neuromuscular coordination and proprioceptive awareness, this immediate representation of segment orientation and symmetry may facilitate awareness and support the internalization of correct motor patterns.

While it is not possible within the current design to isolate the exclusive effect of sensor visualization from traditional coaching input, the consistent improvements across participants suggest that the combination of structured drills and real-time biomechanical feedback may accelerate technique refinement relative to verbal instruction alone. Future controlled studies should aim to disentangle these interacting feedback mechanisms.

### 4.4. Practical Implications for Coaching and Technology Integration

From a practical perspective, integrating wearable sensor systems into routine swimming sessions may offer coaches objective insights into lower-limb mechanics that are otherwise difficult to quantify visually, particularly underwater. In breaststroke, subtle deviations in ankle positioning or symmetry during the recovery phase can significantly affect propulsion. Sensor-assisted monitoring may therefore complement traditional qualitative coaching cues.

The feasibility demonstrated in this pilot study indicates that wearable IMUs can be implemented in ecological training environments without disrupting session structure. For youth swimmers, such technology may support individualized technique monitoring and progression tracking. As wearable devices continue to evolve in miniaturization and algorithmic robustness, their integration into skill-dependent sports such as swimming is likely to expand.

### 4.5. Limitations and Future Research Directions

Despite the promising findings, several limitations must be acknowledged. First, the study involved a small sample size, which is characteristic of exploratory pilot investigations and limits generalizability. The primary objective was feasibility assessment rather than inferential generalization.

Second, although improvements were observed following the intervention, the absence of a control group prevents isolation of the specific contribution of sensor-based feedback from normal training progression effects.

Third, participants received continuous verbal feedback from the coach during the intervention. Therefore, the isolated effect of real-time sensor visualization cannot be fully separated from traditional instructional guidance. Future controlled studies should compare sensor-assisted feedback with verbal-only coaching to clarify the independent contribution of wearable visualization systems.

Finally, the study focused primarily on descriptive performance outcomes and qualitative technique evaluation. Further research should incorporate larger cohorts, longer intervention periods, and quantitative analysis of specific sensor-derived kinematic variables to strengthen the evidence base.

Several additional limitations must be acknowledged. Technique scores were not assessed under blinded conditions, and both evaluators were aware of the intervention timeline, which may have introduced expectancy bias. The potential influence of wearing wearable sensors on hydrodynamics should also be considered. Although the IMUs were compact and securely fixed, their presence may have introduced minimal drag or movement constraint. Furthermore, the small, non-random sample and narrow age range limit generalizability of the findings to swimmers of similar age and proficiency levels.

## 5. Conclusions

This exploratory pilot study examined the feasibility and pre–post changes associated with a sensor-assisted training intervention on breaststroke kick performance in young swimmers. The results indicate that integrating wearable inertial sensors into a structured training program is feasible in a swimming environment and can support improvements in both time-based performance and technical execution of the breaststroke kick.

Consistent reductions in swimming times, together with improved qualitative scores for both the passive and active phases of the kick, suggest enhanced lower-limb coordination, propulsion efficiency, and technique consolidation following the intervention. The use of real-time kinematic feedback provided by wearable sensors appears to facilitate technique awareness and correction, particularly in young swimmers at early stages of technical development.

From a practical perspective, the findings highlight the potential value of sensor-based feedback as a complementary tool to traditional coaching methods. Wearable inertial sensors allow continuous monitoring of movement execution during regular training sessions, enabling more objective assessment and targeted technique refinement without disrupting the training process.

Given the exploratory nature of the study and the limited sample size, the results should be interpreted with caution. However, the observed trends support further investigation using larger cohorts, extended intervention periods, and more detailed quantitative analysis of sensor-derived kinematic variables. Future research may contribute to establishing evidence-based guidelines for the integration of wearable sensor technology into swimming training and performance evaluation.

## Figures and Tables

**Table 1:** Baseline, post-intervention values and paired differences for the 40 m Breaststroke Kick with Kickboard.

Variable	Baseline (Mean ± SD)	Post (Mean ± SD)	Mean Difference (Post–Pre) ± SD	Hedges g (Paired)
40 m Kick with Kickboard (s)	78.30 ± 4.11	69.24 ± 5.06	−9.06 ± 1.69	4.79

**Table 2:** Baseline, post-intervention values and paired differences for the 40 m Breaststroke (no kickboard).

Variable	Baseline (Mean ± SD)	Post (Mean ± SD)	Mean Difference (Post–Pre) ± SD	Hedges g (Paired)
40 m Breaststroke (s)	90.45 ± 1.36	82.28 ± 1.86	−8.17 ± 1.28	5.30

**Table 3:** Baseline, post-intervention values and paired differences for Passive Phase Technique Scores.

Variable	Baseline (Mean ± SD)	Post (Mean ± SD)	Mean Difference (Post–Pre) ± SD	Hedges g (Paired)
Passive Phase Score	6.20 ± 0.84	8.25 ± 0.50	2.05 ± 0.68	2.72

**Table 4:** Baseline, post-intervention values and paired differences for Active Phase Technique Scores.

Variable	Baseline (Mean ± SD)	Post (Mean ± SD)	Mean Difference (Post–Pre) ± SD	Hedges g (Paired)
Active Phase Score	7.98 ± 0.48	8.65 ± 0.57	1.35 ± 0.74	1.54

**Table 5:** Baseline, post-intervention values and paired differences for sensor-derived kinematic variables.

Variable	Baseline (Mean ± SD)	Post-Intervention (Mean ± SD)	Mean Difference (Post–Pre) ± SD	Hedges g (Paired)
Peak ankle dorsiflexion (°)	10.0 ± 2.0	16.2 ± 1.3	6.2 ± 0.84	6.46
Peak external foot rotation (°)	42.8 ± 1.9	50.0 ± 1.9	7.2 ± 0.45	14.45

## Data Availability

The data presented in this study are available on request from the corresponding author. The data are not publicly available due to privacy restrictions involving minor participants and the policies of the participating sports clubs.

## Supplementary Materials

The following supporting information can be downloaded at: https://www.mdpi.com/article/10.3390/s26051691/s1, Supplementary Material S1 provides the detailed weekly structure of the sensor-assisted training program. Supplementary Material S2 contains individual participant technique scores for both raters at baseline and post-intervention.
