# Supplementary material for: Biomechanical Analysis of the Breaststroke Kick in Young Swimmers Using Wearable Inertial Sensors: An Exploratory Pilot Study
Source: Sensors (Basel). 2026 Mar 7;26(5):1691. doi: 10.3390/s26051691 (PMC12987037; doi:10.3390/s26051691)
Supplement: Supplementary file 1 [file sensors-26-01691-s001.zip › sensors-4175678-supplementary.pdf]

Supplementary Material S1 provides the detailed weekly structure of the sensor-assisted training program

S1. Experimental Training Program Overview

The experimental training program was implemented over a five-week period, with one training session per week. During all exercises, participants wore four wearable inertial sensors attached to the lower limbs. In addition to the breaststroke kick – specific drills aimed at technique correction and consolidation, each training session also included general warm-up activities and complementary swimming exercises related to other strokes.

Throughout all training sessions, the KineXYZ application was used in conjunction with the wearable sensors to provide real-time visualization of the executed movements and the joint angles generated during breaststroke kick execution.

The sensors were positioned as follows: one sensor on the right shank, one on the left shank, one on the right foot, and one on the left foot.

S2. Training Session 1

1. **Seated on the floor with arms supported behind the body**, execution of breaststroke leg movements. Sensors attached to the feet and shanks. *4 sets × 10 repetitions.*
2. **Supine position on a bench or lounger**, with the lower limbs unsupported. With the assistance of a partner, the feet were placed sole-to-sole, then heels-to-heels, followed by execution of the breaststroke kick. Sensors attached to the feet and shanks. *4 sets × 10 repetitions.*
3. **Supine position on a bench or lounger**, lower limbs unsupported. With partner assistance, the feet were positioned heel-to-heel, followed by execution of the breaststroke kick. Sensors attached to the feet and shanks. *4 sets × 10 repetitions.*
4. **Supine position on a bench or lounger**, lower limbs unsupported. Exercise performed without a partner. Feet positioned sole-to-sole, then heels-to-heels, followed by breaststroke kick execution. Sensors attached to the feet and shanks. *4 sets × 10 repetitions.*
5. **Supine position on a bench or lounger**, lower limbs unsupported. Exercise performed without a partner, feet positioned heel-to-heel, executing the breaststroke kick. Sensors attached to the feet and shanks. *4 sets × 10 repetitions.*
6. **Seated on the pool edge with arm support**, exercise performed with a partner located in the water. The partner assisted the participant in executing heel movement toward the pool edge by abducting the knees, with the heels contacting the pool wall. Toes were dorsiflexed toward the shanks, and heels were externally rotated. Sensors attached to the feet and shanks. *4 sets × 10 repetitions.*
7. **Seated on the pool edge with arm support**, legs extended. Exercise performed with partner assistance from the water, guiding correct execution of the breaststroke kick. Sensors attached to the feet and shanks. *4 sets × 10 repetitions.*

S3. Training Session 2

1. **Prone position with the chest supported on the starting block**, imitation of the breaststroke kick. Exercise performed with partner assistance to ensure correct movement execution. Sensors attached to the feet and shanks. *4 sets × 1 min.*
2. **Prone position with the chest supported on the starting block**, imitation of the breaststroke kick without partner assistance. Sensors attached to the feet and shanks. *4 sets × 1 min.*
3. **Prone position at the pool edge**, with the legs submerged and the trunk supported on the pool edge. Feet positioned sole-to-sole, then heels-to-heels, followed by execution of the breaststroke kick. Sensors attached to the feet and shanks. *4 sets × 1 min.*
4. **Prone position at the pool edge**, legs submerged, trunk supported. Feet positioned heel-to-heel, executing the breaststroke kick. Sensors attached to the feet and shanks. *4 sets × 1 min.*
5. **In-water position at the pool edge**, arms extended and trunk submerged. Feet positioned sole-tosole,
then heels-to-heels, executing the breaststroke kick. Sensors attached to the feet and shanks.

4 sets ×2 min.
6. **In-water position at the pool edge**, arms extended and trunk submerged. Feet positioned heel-toheel,
executing the breaststroke kick. Sensors attached to the feet and shanks.
4 sets ×2 min.
7. **Prone position at the pool edge with arms extended**, executing the breaststroke kick with knee contact against the pool wall during the recovery phase. Sensors attached to the feet and shanks.
4 sets ×1 min.

S4. Training Session 3

1. **In-water position at the pool edge**, arms extended and trunk submerged, feet positioned heel-toheel,
executing the breaststroke kick. Sensors attached to the feet and shanks. *2 sets ×2 min.*
2. **Prone position at the pool edge with arms extended**, executing the breaststroke kick with knee contact against the pool wall during the recovery phase. Sensors attached to the feet and shanks.
2 sets ×2 min.
3. **With arms extended on a kickboard and head above water**, feet positioned sole-to-sole, then heels-to-heels, executing the breaststroke kick. Sensors attached to the feet and shanks.
4 sets ×40 m.
4. **With arms extended on a kickboard and head above water**, feet positioned heel-to-heel, executing the breaststroke kick. Sensors attached to the feet and shanks. *2 sets ×40 m.*
5. **With arms extended on a kickboard and head above water**, executing the breaststroke kick freely. Sensors attached to the feet and shanks. *2 sets ×40 m.*
6. **With arms extended on a kickboard and head submerged**, feet positioned sole-to-sole, then heelsto-
heels, executing the breaststroke kick with breathing achieved by lifting the head above water. Sensors attached to the feet and shanks. *2 sets ×40 m.*
7. **With arms extended on a kickboard and head submerged**, feet positioned heel-to-heel, executing the breaststroke kick with breathing achieved by lifting the head above water. Sensors attached to the feet and shanks. *2 sets ×40 m.*

S5. Training Session 4

1. **Prone position at the pool edge with arms extended**, executing the breaststroke kick. Sensors attached to the feet and shanks. *2 sets ×2 min.*
2. **Prone position at the pool edge with arms extended**, executing the breaststroke kick with a training stick placed between the legs. *2 sets ×2 min.*
3. **With arms extended on a kickboard and head above water**, executing the breaststroke kick. Sensors attached to the feet and shanks. *2 sets ×40 m.*
4. **With arms extended on a kickboard and head submerged**, executing the breaststroke kick with breathing achieved by lifting the head above water. Sensors attached to the feet and shanks.
2 sets ×40 m.
5. **Swimming with a kickboard and head above water**, executing the breaststroke kick with a training stick placed between the legs. Sensors attached to the feet and shanks. *2 sets ×40 m.*
6. **Swimming with arms extended forward and head submerged**, executing the breaststroke kick with a training stick placed between the legs. Sensors attached to the feet and shanks.
2 sets ×40 m.
7. **Swimming with a kickboard**, executing the breaststroke kick with emphasis on leg separation. Sensors attached to the feet and shanks. *2 sets ×40 m.*

S6. Training Session 5

1. **Prone position at the pool edge with arms extended**, executing the breaststroke kick with strong leg propulsion. Sensors attached to the feet and shanks. *2 sets ×3 min.*
2. **At the pool edge with feet placed on the wall**, performing powerful leg push-offs with arms extended. Sensors attached to the feet and shanks. *2 sets ×3 min.*
3. **Swimming with arms supported on a kickboard and head above water**, emphasizing push-off

from the pool wall. Sensors attached to the feet and shanks. *2 sets × 40 m.*
4. **Swimming with arms supported on a kickboard and head submerged**, executing the breaststroke kick with strong propulsion and breathing achieved by lifting the head. Sensors attached to the feet and shanks. *2 sets × 40 m.*
5. **Swimming with a kickboard and head above water**, executing the breaststroke kick. Sensors attached to the feet and shanks. *2 sets × 40 m.*
6. **Swimming with arms extended forward**, executing the breaststroke kick with breathing performed at each propulsion phase. Sensors attached to the feet and shanks. *2 sets × 40 m.*
7. **Full breaststroke swimming without a kickboard**. Sensors attached to the feet and shanks. *2 sets × 40 m.*

Supplementary Material S2 contains individual participant technique scores for both raters at baseline and post-intervention.

Table S2.1 – Passive phase individual scores

	Subject	Baseline		Post-Intervention	
		Coach	Student	Coach	Student
Passive Phase	Subject 1	7	8	9	9
	Subject 2	6	7	7	9
	Subject 3	5	5	8	8
	Subject 4	7	6	8,5	9
	Subject 5	5	6	7	8

Table S2.2 – Active phase individual scores

	Subject	Baseline		Post-Intervention	
		Coach	Student	Coach	Student
Active Phase	Subject 1	8	8,5	9,5	9
	Subject 2	6	6	8	9
	Subject 3	7	8	8	8
	Subject 4	7	7,5	9	8,5
	Subject 5	8	7	8,5	9
